# Favorable Effect of Anti-TNF Therapy on Insulin Sensitivity in Nonobese, Nondiabetic Patients with Inflammatory Bowel Disease

**DOI:** 10.1155/2018/6712901

**Published:** 2018-03-05

**Authors:** Stavroula A. Paschou, Fotios Kothonas, Apostolos Lafkas, Alexandros Myroforidis, Vasiliki Loi, Thomais Terzi, Olympia Karagianni, Androniki Poulou, Konstantinos Goumas, Andromachi Vryonidou

**Affiliations:** ^1^Department of Endocrinology and Diabetes, Hellenic Red Cross Hospital, Athens, Greece; ^2^Department of Gastroenterology, Hellenic Red Cross Hospital, Athens, Greece

## Abstract

**Background:**

The aim of this study was to investigate the effect of anti-TNF therapy on glucose and lipid metabolism in nondiabetic, nonobese patients with inflammatory bowel disease (IBD).

**Patients and Methods:**

We studied 44 patients with IBD, without a known history of diabetes. Three of the patients were diagnosed with overt diabetes and were excluded. Eighteen of the remaining patients (9 M/9 F, 33.6 ± 8.8 years) were on anti-TNF therapy for longer than 1 year, while 23 patients (16 M/7 F, 38.7 ± 12.5 years) were treated with aminosalicylates (AMSs). Twelve of the patients from the second group were then treated with anti-TNF and reassessed 6 months later. Fasting glucose, insulin, c-peptide, HbA1c, lipid, CRP, and fibrinogen levels were determined, and HOMA-IR index was calculated in all patients.

**Results:**

Patients from the two therapy groups were matched for age and BMI and were not obese. We did not find any differences between patients from the two therapy groups regarding fasting glucose, c-peptide, HbA1c, total cholesterol, HDL, LDL, triglycerides, CRP, and HOMA-IR index. In patients who were treated for 6 months with anti-TNF, a statistically significant decrease in insulin (before 15.5 ± 5.9 versus after 9.9 ± 2.9 *μ*IU/ml, *p* = 0.042) and c-peptide (before 2.4 ± 1 versus after 1.3 ± 0.4 ng/ml, *p* = 0.030) levels as well as the HOMA-IR index (before 4.2 ± 1.9 versus after 2.2 ± 0.9, *p* = 0.045) was observed, without any changes in weight, BMI, glucose, HbA1c, lipid, CRP, and fibrinogen levels.

**Conclusion:**

Anti-TNF therapy exerts a favorable effect on insulin sensitivity, while it has no effect on lipid levels in nondiabetic, nonobese patients with inflammatory bowel disease.

## 1. Introduction

Inflammatory bowel disease (IBD) is an immune-mediated disorder of the intestine, encompassing two different clinical entities, Crohn's disease and ulcerative colitis. These diseases share some clinical features but also present distinct characteristics regarding both histopathology and topography [1]. As tumor necrosis factor alpha (TNF*α*) plays a central role in the inflammatory process of IBD, many of these patients with active disease are treated with anti-TNF agents [2, 3].

Patients with autoimmune diseases often present insulin resistance, dyslipidemia, type 2 diabetes, and increased cardiovascular risk. This has been reported in rheumatoid arthritis and psoriasis [4, 5]. In patients with IBD, insulin resistance is also present, but the presence of increased risk for type 2 diabetes and cardiovascular disease has not been documented yet [6, 7]. Inflammation and insulin resistance are closely linked, and inflammatory cytokines such as tumor necrosis factor alpha (TNF*α*) may inhibit insulin signaling and promote insulin resistance [8]. Specifically, TNF*α* induces phosphorylation of insulin receptor substrate proteins at serine residues instead of tyrosine ones. As phosphorylation at tyrosine residues is essential for the initiation of intracellular signaling cascade and normal insulin action, TNF*α* leads to insulin resistance [9–11]. Anti-TNF therapy reduces activity and severity of inflammatory autoimmune diseases. Furthermore, such therapy has resulted in the reduction of cardiovascular disease events. This reduction is the result not only of the reduction of the inflammation itself but also of the improvement in insulin sensitivity [12–15].

The aim of this study was to investigate the effect of anti-TNF therapy on glucose and lipid metabolism in nondiabetic, nonobese patients with IBD.

## 2. Patients and Methods

### 2.1. Patients

Two substudies were performed, one observational and one prospective interventional. The study was conducted at the Department of Gastroenterology and the Department of Endocrinology of Hellenic Red Cross Hospital in Athens, Greece. Research has been approved by the Institutional Review Board of the Hellenic Red Cross Hospital. Investigations were conducted according to the principles of the Declaration of Helsinki. All participants provided written informed consent.

We included in our study 44 patients with IBD (25 M/19 F, 36.4 ± 11 (19–64) years old, 29 with Crohn's disease, and 15 with ulcerative colitis), without a known history of diabetes and other severe or chronic clinical conditions. Three of the patients were diagnosed with overt diabetes and were excluded from the final statistical analysis. Eighteen patients (9 M/9 F, 33.6 ± 8.8 years) were on anti-TNF therapy for more than 1 year, while the other 23 patients (16 M/7 F, 38.7 ± 12.5 years) were treated with aminosalicylates (AMSs). Twelve of the patients from the second group were then treated with anti-TNF agents (infliximab and adalimumab) and reassessed after 6 months.

### 2.2. Methods

A medical doctor (gastroenterologist or endocrinologist) took the medical history and performed physical examination in each participant. Weight, height, and waist circumference were recorded. Morning blood samples were drawn after an overnight fast from all patients. Inflammation markers including high-sensitivity CRP levels and fibrinogen levels were evaluated by nephelometry (BN Prospec System, Siemens, and Diagnostica Stago), while glucose, insulin, c-peptide, HbA1c, and lipids were determined by assays already described by our group [16]. HOMA-IR (homeostatic model assessment insulin resistance) index was calculated for the evaluation of insulin resistance in all patients. The mathematic model for HOMA-IR is as follows: glucose × insulin/405 (glucose in mg/dl).

### 2.3. Statistical Analysis

Results are presented as the mean ± SD. The Statistical Package for the Social Sciences (SPSS 16.0 Inc., Chicago, IL, USA) was used for all statistical analyses. A *p* value of <0.05 was considered statistically significant. Kolmogorov-Smirnov test was used for the evaluation of the distribution of continuous parameters. Differences between groups were tested using the independent *t*-test or the nonparametric Mann–Whitney *U* test. Differences before and after intervention were tested using the paired *t*-test.

## 3. Results

The characteristics of the patients from the two therapy groups are presented in Table 1. Patients from two groups were matched for age (anti-TNF 33.6 ± 8.8 years versus AMSs 38.7 ± 12.5 years, *p* > 0.05) and BMI (anti-TNF 23.3 ± 3.4 versus AMSs 23.1 ± 1.7, *p* > 0.05) and were not obese. We did not find any statistical differences between patients from the two therapy groups in the levels of fasting glucose (anti-TNF 88 ± 10.7 versus AMSs 93.4 ± 14.9 mg/dl, *p* > 0.05), insulin (anti-TNF 10.9 ± 7.9 versus AMSs 12.1 ± 6.6 *μ*IU/ml, *p* > 0.05), c-peptide (anti-TNF 1.9 ± 0.9 versus AMSs 2.2 ± 1.4 ng/ml, *p* > 0.05), HbA1c (anti-TNF 5.2 ± 0.3 versus AMSs 5.3 ± 0.4%, *p* > 0.05), total cholesterol (anti-TNF 168.6 ± 32.7 versus AMSs 162.8 ± 34.3 mg/dl, *p* > 0.05), HDL (anti-TNF 57.5 ± 15.7 versus AMSs 53.8 ± 20.3 mg/dl, *p* > 0.05), LDL (anti-TNF 95.8 ± 28.7 versus AMSs 90.7 ± 24.4 mg/dl, *p* > 0.05), triglycerides (anti-TNF 75.8 ± 37.6 versus AMSs 90.8 ± 61.3 mg/dl, *p* > 0.05), CRP (anti-TNF 3 ± 5.4 versus AMSs 4.9 ± 6.1, *p* > 0.05), and the HOMA-IR index (anti-TNF 2.77 ± 2 versus AMSs 3.1 ± 1.9, *p* > 0.05).

Twelve of the patients from the second group were then treated with anti-TNF and reassessed after 6 months on treatment. The comparisons before and after intervention with anti-TNF are presented in Table 2. A statistically significant decrease in insulin (before 15.5 ± 5.9 versus after 9.9 ± 2.9 *μ*IU/ml, *p* = 0.042) and c-peptide (before 2.4 ± 1 versus after 1.3 ± 0.4 ng/ml, *p* = 0.030) levels as well as in the HOMA-IR index (before 4.2 ± 1.9 versus after 2.2 ± 0.9, *p* = 0.045) was observed, without any statistically significant changes in weight, BMI, glucose, HbA1c, lipid, and CRP levels (*p* > 0.05 in all comparisons).

## 4. Discussion

The aim of this study was to investigate the effect of anti-TNF therapy on glucose and lipid metabolism in patients with IBD, and for that reason, we performed two substudies, one observational and one prospective interventional. The prospective interventional substudy proved that anti-TNF therapy has a favorable effect on insulin sensitivity in nondiabetic, nonobese patients with inflammatory bowel disease. Specifically, we found a significant reduction in insulin, c-peptide levels, and HOMA-IR after six months of treatment with anti-TNF. This improvement in insulin sensitivity was observed without significant changes in BMI, lipid levels, and inflammation markers.

Our results are in accordance with few previous studies that showed significant improvement of insulin sensitivity after anti-TNF therapy in patients with rheumatoid arthritis, psoriasis, and ankylosing spondylitis, especially in subgroups with higher insulin resistance [12–15, 17–20]. A following more detailed study, including patients with active rheumatoid arthritis and high insulin resistance, confirmed these findings while it also proved restoration of mediators' phosphorylation in the insulin signaling cascade, after 12 weeks of treatment with anti-TNF agents [21]. Furthermore, another retrospective study in which anti-TNF agents were used in larger therapeutic doses for up to 10 years in a very small group of patients with rheumatoid arthritis and Crohn's disease resulted in significantly better glycemic control in patients with coexistent type 2 diabetes [22].

In patients with IBD, the role of TNF*α* in glucose metabolism is still unclear. In two studies including patients with active IBD, increased insulin resistance and impaired beta cell function were found [6, 7], while in the remission state normal insulin sensitivity was reported [23]. To our knowledge, our study is the first one conducted in nondiabetic, nonobese patients with inflammatory bowel disease which showed a reduction of insulin resistance after anti-TNF therapy. Another relevant study of shorter duration did not find any alterations in insulin resistance in IBD patients, though this could be related to a significant increase in BMI in all patients [24]. Similar was the result of a study including patients with active rheumatoid arthritis who showed marked insulin resistance, but this was not influenced by anti-TNF therapy despite a reduction in systemic inflammation after treatment [25].

Previous studies performed in various populations of patients with inflammatory autoimmune diseases indicated an increased risk for insulin resistance, dyslipidemia, type 2 diabetes, and atherosclerosis [26–28]. Recent evidence from a large cohort study from the UK suggested that cardiovascular risk is positively correlated with the severity of inflammation [29]. Several studies have reported that inflammation, insulin resistance, and carotid intima-media thickness are increased in patients with IBD [6, 30, 31]. Patients with IBD, especially those with Crohn disease, are young in age at the diagnosis and present lower BMI from malnutrition, and corticosteroids are used in a lesser extent for disease management, compared to patients with other chronic inflammatory diseases [29, 32–34]. Moreover, lipid levels, which represent an additional important cardiovascular risk factor, are normal or low in these patients [35]. In our study, lipid levels were normal and no changes were noted before and posttreatment with anti-TNF. A recent meta-analysis encompassing 766 patients with rheumatoid arthritis concluded that anti-TNF treatment had no significant effect on lipid levels, as well as the atherogenic index [36].

Nevertheless, the reduction of insulin resistance with or without the reduction of inflammation may have clinical implications for the reduction of cardiovascular event risks in patients with IBD. Such a reduction has already been reported in patients with rheumatoid arthritis [37, 38]. Furthermore, our results highlight possible clinical implications for the use of anti-inflammatory agents in our therapeutic armamentarium for metabolic syndrome and type 2 diabetes. If type 2 diabetes is a clinical state of inflammation with high levels of cytokines such as TNF*α* [9–11, 39–41] and these levels can be reduced by certain anti-inflammatory drugs [2, 3], why these cannot be used as another component of our treatment strategy for this common disease? Of course, well-designed studies with specific patient populations are needed before such practice can be introduced, but the theoretical background is sound and our study adds on that.

The fact that we did not find any statistically significant differences for these parameters in the observational substudy is a matter of statistics, in our opinion. In the order of differences to be shown by independent statistical analyses, larger numbers of participants are needed compared to paired statistical analyses. Furthermore, the interventional nature of the second substudy renders our findings really sound. Of course, a larger sample size in both substudies would be ideal. However, it is difficult in everyday clinical practice to find patients fulfilling such criteria. This is also reflected by the relatively small sample size of previous studies, including patients with rheumatoid arthritis, psoriasis, and ankylosing spondylitis [12–15, 18, 20, 21].

In conclusion, this study provides evidence that anti-TNF therapy has a favorable effect on insulin sensitivity and no effect on lipid levels in nondiabetic, nonobese patients with inflammatory bowel disease. These data indicate that endogenous TNF*α* may be a causative factor for insulin resistance and type 2 diabetes, while these highlight possible clinical implications for the use of anti-inflammatory agents in the therapeutic armamentarium for metabolic syndrome and type 2 diabetes.

## Figures and Tables

**Table 1:** Characteristics of patients from the two therapy groups (results presented as mean ± SD).

	AMSs (*n* = 23)	Anti-TNF (*n* = 18)	*p* value
Age (years)	38.7 ± 12.5	33.6 ± 8.8	ns
Gender	16 M/7 F	9 M/9 F	ns
BMI (kg/m^2^)	23.1 ± 1.7	23.3 ± 3.4	ns
Fasting glucose (mg/dl)	93.4 ± 14.9	88 ± 10.7	ns
Insulin (*μ*IU/ml)	12.1 ± 6.6	10.9 ± 7.9	ns
C-peptide (ng/ml)	2.2 ± 1.4	1.9 ± 0.9	ns
HbA1c (%)	5.3 ± 0.4	5.2 ± 0.3	ns
Cholesterol (mg/dl)	162.8 ± 34.3	168.6 ± 32.7	ns
HDL (mg/dl)	53.8 ± 20.3	57.5 ± 15.7	ns
LDL (mg/dl)	90.7 ± 24.4	95.8 ± 28.7	ns
Triglycerides (mg/dl)	90.8 ± 61.3	75.8 ± 37.6	ns
CRP (mg/l)	4.9 ± 6.1	3 ± 5.4	ns
HOMA-IR	3.1 ± 1.9	2.77 ± 2	ns

AMSs: aminosalicylates; BMI: body mass index; CRP: C-reactive protein; HDL: high-density lipoprotein; HOMA-IR: homeostatic model assessment insulin resistance; LDL: low-density lipoprotein; TNF*α*: tumor necrosis factor alpha.

**Table 2:** Comparisons before and after intervention with anti-TNF treatment (results presented as mean ± SD).

	Baseline (*n* = 12)	6 months after anti-TNF (*n* = 12)	*p* value
Age (years)	43.1 ± 10.8		
Gender	7 M/5 F		
BMI (kg/m^2^)	23.8 ± 1.4	24.1 ± 1.5	ns
Fasting glucose (mg/dl)	103 ± 5.9	96.5 ± 10.2	ns
Insulin (*μ*IU/ml)	15.5 ± 5.9	9.9 ± 2.9	0.042
C-peptide (ng/ml)	2.4 ± 1	1.3 ± 0.4	0.030
HbA1c (%)	5.4 ± 0.3	5.2 ± 0.3	ns
Cholesterol (mg/dl)	176.8 ± 35.2	167.6 ± 36.8	ns
HDL (mg/dl)	49.6 ± 18.6	51.4 ± 11.9	ns
LDL (mg/dl)	98.5 ± 26.7	94.7 ± 36.2	ns
Triglycerides (mg/dl)	138.9 ± 62.5	114.2 ± 57.5	ns
CRP (mg/l)	6.1 ± 8	5.2 ± 8.1	ns
HOMA-IR	4.2 ± 1.9	2.2 ± 0.9	0.045

AMSs: aminosalicylates; BMI: body mass index; CRP: C-reactive protein; HDL: high-density lipoprotein; HOMA-IR: homeostatic model assessment insulin resistance; LDL: low-density lipoprotein; TNF*α*: tumor necrosis factor alpha.

## Abbreviations

AMSs: Aminosalicylates
BMI: Body mass index
CRP: C-reactive protein
CVD: Cardiovascular disease
HDL: High-density lipoprotein
HOMA-IR: Homeostatic model assessment insulin resistance
IBD: Inflammatory bowel disease
LDL: Low-density lipoprotein
TNF*α*: Tumor necrosis factor alpha.
